# Highly sensitive real-time PCR for specific detection and quantification of *Coxiella burnetii*

**DOI:** 10.1186/1471-2180-6-2

**Published:** 2006-01-19

**Authors:** Silke R Klee, Judith Tyczka, Heinz Ellerbrok, Tatjana Franz, Sonja Linke, Georg Baljer, Bernd Appel

**Affiliations:** 1 Robert Koch-Institut, Centre for Biological Safety, Nordufer 20, 13353 Berlin, Germany; 2 Institute for Hygiene and Infectious Diseases of Animals, Justus Liebig University of Giessen, Frankfurter Str. 85-89, 35392 Giessen, Germany; 3 Federal Institute for Risk Assessment, Diedersdorfer Weg 1, 12277 Berlin, Germany

## Abstract

**Background:**

*Coxiella burnetii*, the bacterium causing Q fever, is an obligate intracellular biosafety level 3 agent. Detection and quantification of these bacteria with conventional methods is time consuming and dangerous. During the last years, several PCR based diagnostic assays were developed to detect *C. burnetii *DNA in cell cultures and clinical samples. We developed and evaluated TaqMan-based real-time PCR assays that targeted the singular *icd *(isocitrate dehydrogenase) gene and the transposase of the *IS1111a *element present in multiple copies in the *C. burnetii *genome.

**Results:**

To evaluate the precision of the *icd *and *IS1111 *real-time PCR assays, we performed different PCR runs with independent DNA dilutions of the *C. burnetii *Nine Mile RSA493 strain. The results showed very low variability, indicating efficient reproducibility of both assays. Using probit analysis, we determined that the minimal number of genome equivalents per reaction that could be detected with a 95% probability was 10 for the *icd *marker and 6.5 for the *IS *marker. Plasmid standards with cloned *icd *and *IS1111 *fragments were used to establish standard curves which were linear over a range from 10 to 10^7 ^starting plasmid copy numbers. We were able to quantify cell numbers of a diluted, heat-inactivated *Coxiella *isolate with a detection limit of 17 *C. burnetii *particles per reaction. Real-time PCR targeting both markers was performed with DNA of 75 different *C. burnetii *isolates originating from all over the world. Using this approach, the number of *IS1111 *elements in the genome of the Nine Mile strain was determined to be 23, close to 20, the number revealed by genome sequencing. In other isolates, the number of *IS1111 *elements varied widely (between seven and 110) and seemed to be very high in some isolates.

**Conclusion:**

We validated TaqMan-based real-time PCR assays targeting the *icd *and *IS1111 *markers of *C. burnetii*. The assays were shown to be specific, highly sensitive and efficiently reproducible. Cell numbers in dilutions of a *C. burnetii *isolate were reliably quantified. PCR quantification suggested a high variability of the number of *IS1111 *elements in different *C. burnetii *isolates, which may be useful for further phylogenetic studies.

## Background

*Coxiella burnetii *is the causative agent of Q fever, a zoonosis that occurs worldwide and infects a variety of different animals, including domestic mammals like cattle and sheep. Whereas animals in general show no clinical signs of infection except occasional abortions, *C. burnetii *can cause serious illness in humans, where infections usually occur via aerosols. Acute disease often presents as a self-limiting influenza-like illness with fever and headaches, but severe cases with atypical pneumonia or hepatitis may occur. The disease can become chronic with life-threatening endocarditis as the most frequent clinical manifestation that requires long lasting antibiotic treatment [1]. Although an obligate intracellular organsim, the bacterium is very resistant to environmental conditions due to extracellular spore-like forms, and even a single organism can produce disease. Because of its widespread availability, environmental stability and low infective dose, *C. burnetii *is considered a potential bioterrorist agent and is classified as a group B agent by the Centers for Disease Control and Prevention in Atlanta, USA [2].

*C. burnetii *is a slow growing bacterium that can be cultivated in embryonated eggs or eukaryotic cell culture, which is time consuming and must be performed in biosafety level 3 laboratories. Antigen detection of bacteria by capture ELISA or direct immunofluorescence is difficult and has relatively high detection limits. Therefore, diagnosis is still mainly based on serological methods like indirect immunofluorescence, complement fixation or ELISA, with the disadvantage of delayed diagnosis because specific antibodies appear only one to two weeks after infection [3].

During the last years, several PCR based diagnostic assays were developed to detect *C. burnetii *DNA in cell cultures and clinical samples. These assays used conventional PCR [4-8], nested PCR [9-12] or real-time PCR conditions with LightCycler [13-15], SYBR Green [16] or TaqMan chemistry [17]. The target sequences of the assays originated from singular chromosomal genes like *com1 *or *htpB*, on plasmids (QpH1, QpRS) or on the transposase gene of insertion element *IS1111 *[18] that is present in 20 copies in the genome of the *C. burnetii *Nine Mile RSA493 strain [19]. Due to the multicopy number of the *IS1111 *element, the corresponding PCR is very sensitive. However, quantification of cells cannot be performed based on PCR of the *IS *element, because the numbers of *IS1111 *elements present in different *Coxiella *isolates are not known.

The prerequisite for a diagnostic PCR is a target sequence that is specific for *C. burnetii *to exclude false positive results with other organisms and that is conserved and present in all *C. burnetii *isolates to prevent false negative reactions. The PCR assays mentioned before were usually evaluated with relatively small numbers of characterized isolates or with uncharacterized clinical samples, though it should be noted that most importance was attached on sensitivity of the assay, whereas the suitability of the assays for a great panel of different isolates was less relevant.

The *icd *gene for the isocitrate dehydrogenase was sequenced in 19 strains and shown to be conserved [20]. We used a fragment of this gene as target for real-time TaqMan PCR based on TaqMan chemistry. In addition, we performed a real-time PCR assay based on a fragment of the transposase gene of the *IS *element *IS1111a*. Both assays were validated for specificity and sensitivity, and suitability of the *icd *assay for the quantification of *Coxiella *cell numbers was shown. As the exact number of *IS *elements is only known for the sequenced genome of the Nine Mile strain, we examined the number of *IS1111 *elements per genome, or per *icd *copy, respectively, in a large panel of *Coxiella *isolates of worldwide origin.

## Results and discussion

### Evaluation of the specificity of the real-time PCR assays

To determine whether false positive reactions occurred in real-time PCR assays with the *icd *and *IS1111 *markers, PCR was performed with DNA of the bacterial species listed in the Methods section. Based on the sequence of its 16S rRNA, *C. burnetii *is classified into the order Legionellales, with *Legionella *spp. and *Francisella *spp. as nearest phylogenetic neighbours [1]. Both for these related species and for all other species tested, the PCR was negative, confirming the specificity of both targets.

### Determination of precision and detection limit of the assays

Based on the measured DNA concentration (29 ng/μl) and the length of the published sequence of the *C. burnetii *Nine Mile genome (1,995,275 bp), the theoretical number of genome equivalents (GE) was calculated to be 1.3 × 10^7 ^GE per μl. This corresponds to 2.6 × 10^8 ^*IS1111 *elements per μl for the Nine Mile strain (20 per genome). To determine the precision of the *icd *and *IS *real-time PCR assays, *C*_*t *_(threshold cycle) values for eight replicates of tenfold dilutions of purified *C. burnetii *Nine Mile genomic DNA were measured (Table 2). The results represent independent dilution series and different PCR runs. The mean *C*_*t *_values, standard deviation, and percent CV (coefficient of variation) were calculated for each dilution. The results showed low variability, with CVs ranging from 1.3 to 1.9 % for the *icd *target and 1.1 to 1.6 % for the *IS *target, indicating efficient reproducibility of both assays. Standard curves drawn from the copy numbers and mean *C*_*t *_values shown in Table 2 had slopes of -3.687 for the *icd *curve and -3.527 for the *IS *curve (data not shown), indicating PCR efficiencies of approximately 90 % for both targets (*E *= 10^-1/*s *^- 1, where *E *is the run efficiency and *s *is the slope of the standard curve).

**Figure 1:**
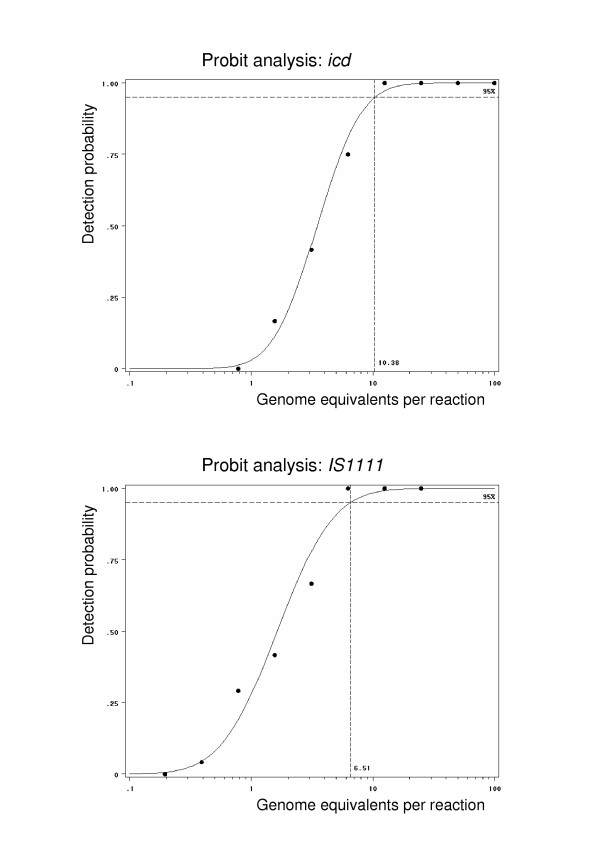
**Determination of detection limits for the *icd *and *IS1111 *assays**. The graphs show curves determined by probit analysis for real-time PCR assays targeting the *icd *and *IS1111a *sequences of *C. burnetii*. With the respective targets, 10 and 6.5 genome equivalents per reaction can be detected with a probability of 95 %.

Determination of the detection limit by probit analysis was performed with DNA of the *C. burnetii *Nine Mile strain. For the singular *icd *marker, detection of 100 to 0.75 GE/reaction was tested by PCR. For the *IS1111 *marker, where 20 copies are expected per genome, lower concentrations from 25 to 0.2 GE/reaction, or 500 to 4 copies of the *IS1111 *element, respectively, were tested. Each PCR was repeated three times with eight replicates for each concentration. The minimal number of genome equivalents per reaction that could be detected with a 95 % probability by real-time PCR was 10 when the *icd *marker was used (Fig. 1). With the *IS1111 *marker, 6.5 genome equivalents per reaction were detected with 95 % probability (Fig. 1), corresponding to 130 copies of the target gene. Detection of lower *IS1111 *copy numbers was possible, as mentioned below for plasmid standards, but less reproducible. PCR products of the *icd *and *IS1111 *assays were analysed on agarose gels and showed the expected single bands of 76 bp and 295 bp, respectively.

### Quantification using plasmid standard curves

Tenfold serial dilutions of plasmids with cloned *icd *and *IS1111 *fragments were used to establish standard curves for each PCR run. For both markers, the quantification was linear over a range of 10 to 10^7 ^starting plasmid copy numbers, and the detection limit was ten copies per reaction (data not shown).

To assess whether the number of *icd *and *IS1111 *copies per genome could be sufficiently calculated by using standard curves derived from plasmid standards, PCR assays for both targets were performed with tenfold serial dilutions of *C. burnetii *Nine Mile DNA and plasmid standards. The results are shown in Table 3. Especially for lower DNA concentrations, the theoretical numbers of *icd *and *IS1111 *copies (calculated from genome size and DNA concentration as shown before) corresponded quite well to the respective copy numbers determined experimentally.

### Determination of *Coxiella *cell numbers by real-time PCR

The cell numbers of purified *Coxiella *isolates can be determined by Gimenez stain. To assess whether the cell densities quanitified by real-time PCR were comparable, we performed PCR reactions of heat inactivated isolates targeting the *icd *marker without previous DNA extraction. An exponential dilution series was made from heat inactivated particles of the Nine Mile isolate containing 4.2 × 10^9 ^particles per ml, and 1 μl of each dilution was applied per PCR reaction. Cell numbers were quantified using standard curves derived from diluted plasmid standards. The results are shown in Table 4. Given that only one copy of the chromosome is present per bacterial cell, which can be expected for a slow growing bacterium like *Coxiella*, the number of genome equivalents based on *icd *quantification should be comparable to the number of bacteria. Indeed, the *icd *quantity correlated well with the numbers of coxiellae determined microscopically. The detection limit for real-time PCR was 17 particles per reaction, which is in good agreement with the detection limit for purified *Coxiella *DNA and far below the particle number that can be quantified microscopically.

### Determination of the number of IS elements in 75 different *Coxiella *isolates

Although the measured *icd *and *IS1111 *copy numbers shown in Table 3 exceeded the calculated numbers in some cases, the number of *IS1111 *elements per genome (i.e., per *icd *copy) varied between 13 and 17 for different DNA concentrations, which is close to the published number of 20 *IS1111 *elements for the Nine Mile isolate. Therefore, with this assay, DNA samples of 75 isolates of *C. burnetii *from all over the world were assessed for presence of the *icd *and *IS *markers and the numbers of *IS1111 *elements per genome were calculated. Quantification of *icd *and *IS *markers was based on standard curves obtained from diluted plasmids. Each DNA sample was tested in duplicate in three independent PCR runs targeting both markers except for DNA from the Nine Mile isolate, where six runs were performed.

In a recent study where Q fever patients were examined 12 years after infection, the *IS1111 *element could not be amplified, whereas PCR for other targets was positive [21]. Our results indicated that all isolates contained both the *icd *and the *IS1111 *markers. Different PCR runs resulted in discrepancies of the measured quantities and accordingly, different values and standard deviations for the number of *IS1111 *elements per genome equivalent were obtained (data not shown). For the Nine Mile RSA493 strain the number of *IS *elements was determined to be 23 (± 3.43), which is in good agreement with the number revealed by sequencing. The mean number of *IS1111 *elements per genome varied between seven (isolate J 3) and 110 (isolate Z2534), and between 10 and 30 for the majority of isolates. In French isolates of the related restriction groups 12 to 16 (Table 1), however, the number of *IS1111 *elements was found to be above 30, being highest in strain "Raphael" (around 95). All isolates of restriction group I had numbers below 30 insertion elements, so that for these isolates a correlation of the number of *IS1111 *elements with the restriction group seems likely. In other restriction groups, however, the number of *IS1111 *elements was highly variable. Although the standard deviations were very high for some values, our data suggest that the number of *IS1111 *elements can vary widely between different *C. burnetii *isolates and some isolates seem to contain a very high number of *IS1111 *elements. To further confirm our real-time PCR based quantification, Southern blot analyses should be performed.

Insertion sequences play a major role in determining band pattern differences between isolates produced by methods such as PFGE (pulsed-field gel electrophoresis) in many bacterial species [22]. *C. burnetii *expresses a low degree of genetic heterogeneity among strains by DNA-DNA hybridization. However, *Not *I restriction of total DNA followed by PFGE resulted in the characterization of 20 restriction groups among 80 *C. burnetii *isolates collected worldwide, as indicated in Table 1[1,23,24]. Typing *C. burnetii *based on restriction fragment length polymorphisms of the locations of the *IS1111 *element, like published for the insertion sequence *IS100 *of *Yersinia pestis *[25], may add to the elucidation of the phylogenetic relationship of *Coxiella *isolates. Moreover, the insertion sites of *IS1111 *could be examined by inverse PCR or by a recently described technique, the so called vectorette PCR [26].

So far, our data are too incomplete for judgements on clinical outcome, namely, to find any correlation between the number of *IS1111 *elements and the virulence of an isolate. Nevertheless, it is tempting to speculate that an increased number of *IS *elements in the genome of an isolate could have a deteriorating effect on its fitness, because essential genes might be interrupted by the insertion sequences.

## Conclusion

We validated TaqMan-based real-time PCR assays targeting the singular *icd *gene and the transposase of the *IS1111a *element present in multiple copies in the genome of *C. burnetii*. The assays were evaluated with a variety of other bacterial species and shown to be specific for *C. burnetii*. Dilution series of *C. burnetii *DNA and of plasmids with cloned *icd *and *IS1111 *inserts demonstrated the sensitivity of the assays. Less than 10 genome equivalents per reaction were reproducibly detected. Using the *icd *marker, cell numbers of *C. burnetii *isolates were quantified also at very low cell concentrations. As a first approximation, the combination of both assays was useful to assess the numbers of *IS1111 *elements in 75 *C. burnetii *isolates from all over the world. Our data indicate that the numbers of this insertion element in the different isolates seem to be highly variable. The differences in the content of *IS1111 *elements might be of importance for further phylogentic analyses of *C. burnetii *isolates.

## Methods

### Bacterial strains and growth conditions

The *C. burnetii *isolates used in this study are shown in Table 1. *C. burnetii *bacteria were grown in Buffalo green monkey cell cultures and isolated as described [7]. To determine bacterial concentrations, a defined volume of a diluted suspension was fixed on a slide and stained by the Gimenez method. Bacteria were counted and the concentration of the suspension was calculated.

The following DNA samples from other bacterial species were used as negative controls for PCR: *Legionella pneumophila *(ATCC 33152, JR32 and 130b), *Francisella tularensis *ssp. *novicida *(ATCC 15482) and ssp. *tularensis *(Schu4), *Bacillus subtilis *(DSM 347), *Bacillus anthracis *(UD III-7), *Bacillus cereus *(DSM 31), *Bacillus thuringiensis *(DSM 350), *Bacillus megaterium *(DSM 90), *Bacillus licheniformis *(DSM 13), *Staphylococcus aureus *(DSM 20231), *Streptococcus equi *(ATCC 9528), *Pseudomonas putida *(ATCC 12633), *Pseudomonas aeruginosa *(ATCC 9027), *Pseudomonas fluorescens *(ATCC 49838), *Burkholderia mallei *(RR0053), *Burkholderia pseudomallei *(ATCC 23343), *Burkholderia stabilis *(CCUG 34168), *Burkholderia multivorans *(CCUG 37240), *Yersinia enterocolitica *(O:8 Ye/80), *Yersinia pseudotuberculosis *(DSM 8992), *Yersinia pestis *(Kim), *Brucella melitensis *biotype 1 (16M Weybridge), *Brucella abortus *biotype 1 (544 Weybridge), *Brucella suis *biotype 1 (1330 Weybridge), *Brucella ovis *biotype 1 (63/290 Weybridge), *Klebsiella oxytoca *(CCUG 15788), *Serratia marcescens*, *Proteus mirabilis*, and *Escherichia coli *(DSM 30083). The DNA preparations of *L. pneumophila *were kind gifts from Dr. A. Flieger (NG 5, Robert Koch-Institut).

### DNA extraction

*C. burnetii *isolates were mixed with an equal volume of ATL Tissue Lysis Buffer (Qiagen, Hilden, Germany) and heat inactivated (90°C, 20 min). DNA was extracted from 400 μl of this suspension according to the protocol for Gram-negative bacteria of the DNeasy Tissue Kit (Qiagen) and eluted in 100 μl of AE buffer.

### Primers and probes for real-time PCR

The *icd *assay targets a 76 bp fragment of the *C. burnetii icd *gene.

Primers:

forward, icd-439F = CGTTATTTTACGGGTGTGCCA (439–459)

reverse, icd-514R = CAGAATTTTCGCGGAAAATCA (494–514)

TaqMan probe:

icd-464TM = FAM-CATATTCACCTTTTCAGGCGTTTTGACCGT-TAMRA-T (464–492).

The numbers in brackets show the positions based on the GenBank accession no. AF146284.

The *IS1111 *assay targets a 295 bp fragment of the transposase gene of the *C. burnetii IS1111a *element.

Primers:

forward, Cox-F = GTCTTAAGGTGGGCTGCGTG (219–238)

reverse, Cox-R = CCCCGAATCTCATTGATCAGC (493–513)

TaqMan probe:

Cox-TM = FAM-AGCGAACCATTGGTATCGGACGTT-TAMRA-TATGG (259–287).

The numbers in brackets show the positions based on the GenBank accession no. M80806.

All sequences are given in 5'-3' orientation. Primers and probes were designed using the Primer Express software (Applied Biosystems, Darmstadt, Germany) and purchased from TIB Molbiol (Berlin, Germany).

### Preparation of plasmid standards

The target sequences were amplified by conventional PCR using DNA from *C. burnetii *Nine Mile RSA493 strain as template and with the same primers as for real-time PCR in the case of the *IS1111 *marker and with primers icd-418F (5'-TATGTTTGCCTTAGGCCCGT) and icd-818R (5'-AAGGGCTTTGCTCCAAATTC) in the case of the *icd *marker, for which a 401 bp long amplicon was obtained. Plasmid standards with cloned (TOPO TA Cloning System, Invitrogen, Karlsruhe, Germany) and sequenced inserts were generated by GenExpress (Berlin, Germany). Plasmid preparations were quantified spectrophotometrically, and plasmid copy numbers were calculated. Dilutions of the plasmids were used in real-time PCR reactions to prepare standard curves for quantification of the initial copy numbers.

### PCR assay conditions

Real-time PCR reaction mix consisted of 6.25 μl of Universal Master Mix (Applied Biosystems, Darmstadt, Germany) containing dNUTPs, MgCl_2_, reaction buffer and AmpliTaq Gold DNA polymerase, 300 nM of each primer and 100 nM of fluorescence-labeled TaqMan probe. For most assays, water was added to a final volume of 24 μl, and 1 μl of purified template DNA or heat inactivated *C. burnetii *isolate was used as template. For determination of the *IS1111 *copy numbers in the 75 *C. burnetii *isolates, water was added to a final volume of 15 μl, and 10 μl of 10-fold dilutions of the DNA were used as templates to minimise pipetting errors. All real-time PCR reactions were performed in duplicate in a 7700 Sequence Detection System (Applied Biosystems) as follows: 2 min at 50°C, 10 min at 95°C, 40 cycles at 15 s 95°C and 30 s at 60°C. Data were analyzed with the corresponding software.

### Probit analysis

The number of *C. burnetii *Nine Mile genome equivalents (GE) with a genome size of 1,995,275 bp in a DNA preparation with a concentration of 29 ng/μl was calculated to be 1.3 × 10^7 ^GE/μl. To determine the number of GE that can be detected with a probability of 95 %, eight replicates of serial DNA dilutions from 100 GE/reaction to 0.75 GE/reaction for *icd *or 25 GE/reaction to 0.2 GE/reaction for *IS1111 *were tested in independent PCR reactions performed by different persons. The reaction volume was 1 μl. Each PCR gave a positive or negative result at the concentration tested. The detection probability was obtained by plotting the proportion of positive PCRs observed against the genome equivalents. Statistical analysis was performed using the SAS version 9.1 software.

## Authors' contributions

SRK designed and coordinated the study, drafted the manuscript and participated in performing real-time PCR assays. JT and GB were responsible for the cultivation of the *C. burnetii *isolates, participated in the design of the study and helped to draft the manuscript. HE participated in the design of the study, the evaluation of the PCR assays and helped to draft the manuscript. TF isolated *C. burnetii *DNA and participated in performing PCR assays. SL participated in DNA isolation and evaluation of the PCR assays. BA participated in the design of the study, provided technical and financial support, and helped to draft the manuscript. All authors read and approved the final manuscript.

## Figures and Tables

**Table 1:** Characteristics of *C. burnetii *isolates used in this study

*C. burnetii *Isolate	Restriction Group [23,24]	Geographical Origin
Nine Mile RSA493	I	USA
Balaceanu	I	Romania
Hardthof	I	Germany
Bernard	I	France
CS 1	I	Slovak Republic
CS 3	I	Slovak Republic
CS 4	I	Slovak Republic
CS 5	I	Slovak Republic
CS 6	I	Slovak Republic
CS 7	I	Slovak Republic
CS 8	I	Slovak Republic
CS 9	I	Slovak Republic
CS 10	I	Slovak Republic
CS 11	I	Slovak Republic
CS Dayer	I	Slovak Republic
CS L 35	I	Slovak Republic
CS Poland	I	Poland
J 1	I	Japan
J 3	I	Japan
J 27	I	Japan
Priscilla Q177	IV	USA
Scurry Q217	V	USA
CS S	V	USA
Dugway 5J108-111	VI	USA
Z 3027	VI	Germany
Z 3205a	VI	Germany
Z 3205b	VI	Germany
Z 3351	VI	Germany
Z 3568	VI	Germany
Z 3749	VI	Germany
Z 257	VI	Germany
Boren	1	USA
CS 48	1	Slovak Republic
CS II/Ia	1	Slovak Republic
CS F	1	Slovak Republic
CS Ixodes	1	Russia
CS S1	1	Russia
Florian	1	Slovak Republic
Frankfurt	1	Germany
München	1	Germany
Henzerling	1	Italy
RT 1	1	North Western Russia
RT 3	1	North Western Russia
Gbud	1	Slovak Republic
Geier	1	Romania
Andelfingen	2	Switzerland
Herzberg	2	Greece
CS Z 57	2	Slovak Republic
S 1	2	Sweden
S 4	2	Sweden
Soyta	2	Switzerland
Utvinis	2	Romania
Stanica	2	Romania
Z 3478	2	Germany
Z 3574	2	Germany
Z 4313	2	Germany
Z 4485	2	Germany
Z 104	2	Germany
Z 3464	4	Germany
Z 3567	5	Germany
Brustel	6	France
Z 2534	6	Austria
Z 3055	6	Germany
Z 2775	7	Germany
Brasov	8	Romania
Namibia	9	Namibia
Schperling	11	Kirgisia
Ouaret	12	France
Jaquemot	13	France
Campoy	13	France
Pallier	14	France
Lombardi	15	France
Raphael	16	France
Butin	16	France
Z 349-36/94	unknown	Germany

**Table 2:** Summary of eight different PCR runs performed on eight separate DNA dilution series of the *C. burnetii *Nine Mile RSA493 strain

*icd *marker				*IS1111 *marker			
No. of copies/μl	Mean *C*_*t*_	SD^a^	CV^b ^(%)	No. of copies/μl	Mean *C*_*t*_	SD^a^	CV^b ^(%)

1.3 × 10^7^	15.81	0.23	1.5	2.6 × 10^8^	15.70	0.25	1.6
1.3 × 10^6^	18.64	0.25	1.3	2.6 × 10^7^	17.63	0.29	1.6
1.3 × 10^5^	22.08	0.33	1.5	2.6 × 10^6^	21.37	0.30	1.4
1.3 × 10^4^	25.93	0.46	1.8	2.6 × 10^5^	25.15	0.27	1.1
1.3 × 10^3^	29.63	0.58	1.9	2.6 × 10^4^	28.76	0.36	1.2
1.3 × 10^2^	33.51	0.60	1.8	2.6 × 10^3^	32.52	0.48	1.5
1.3 × 10^1^	37.79	0.49	1.3	2.6 × 10^2^	36.39	0.51	1.4

^a^SD, standard deviation of eight replicates

^b^CV, coefficient of variation

**Table 3:** PCR quantification of DNA dilutions of the *C. burnetii *Nine Mile RSA493 strain. The measurements were performed in duplicate; mean values are shown.

	Calculated Values^a^		Measured Values^b^		
DNA conc [pg/μl]	*icd*	*IS1111*	*icd*	*IS1111*	*IS *per genome^c^

2900	1.3 × 10^6^	2.6 × 10^7^	4.5 × 10^6^	5.9 × 10^7^	13.1
290	1.3 × 10^5^	2.6 × 10^6^	5.2 × 10^5^	8.1 × 10^6^	15.6
29	1.3 × 10^4^	2.6 × 10^5^	3.9 × 10^4^	5.0 × 10^5^	12.8
2.9	1.3 × 10^3^	2.6 × 10^4^	2.8 × 10^3^	3.5 × 10^4^	12.5
0.29	1.3 × 10^2^	2.6 × 10^3^	2.3 × 10^2^	2.6 × 10^3^	11.3
2.9 × 10^-2^	1.3 × 10^1^	2.6 × 10^2^	1.5 × 10^1^	2.6 × 10^2^	17.3

^a^Number of target copies based on DNA concentration and genome length.

^b^Number of target copies based on PCR quantification using plasmid standards.

^c^Calculated as *IS1111 *measured per *icd *measured.

**Table 4:** Comparison of microscopical and PCR-based determination of *Coxiella *cell numbers. The PCR measurements were performed in duplicate; mean values are shown.

*Coxiella *particles per μl	
Determined microscopically^a^	Quantified by PCR^b^
4.2 × 10^6^	5.00 × 10^6^
4.2 × 10^5^	3.05 × 10^5^
4.2 × 10^4^	3.15 × 10^4^
4.2 × 10^3^	2.45 × 10^3^
4.2 × 10^2^	1.45 × 10^2^
4.2 × 10^1^	1.70 × 10^1^
4.2 × 10^0^	0

^a^Cell numbers were only counted from undiluted sample.

^b^Real-time PCR targeting the *icd *marker.
